# AFP-Inhibiting Fragments for Drug Delivery: The Promise and Challenges of Targeting Therapeutics to Cancers

**DOI:** 10.3389/fcell.2021.635476

**Published:** 2021-04-08

**Authors:** Bo Lin, Xu Dong, Qiujiao Wang, Wei Li, Mingyue Zhu, Mengsen Li

**Affiliations:** ^1^Hainan Provincial Key Laboratory of Carcinogenesis and Intervention, Hainan Medical College, Haikou, China; ^2^Institution of Tumor, Hainan Medical College, Haikou, China

**Keywords:** AFP molecular structure, AFP inhibiting fragments, drug delivery, targeted cancer therapy, drug design

## Abstract

Alpha fetoprotein (AFP) plays a key role in stimulating the growth, metastasis and drug resistance of hepatocellular carcinoma (HCC). AFP is an important target molecule in the treatment of HCC. The application of AFP-derived peptides, AFP fragments and recombinant AFP (AFP-inhibiting fragments, AIFs) to inhibit the binding of AFP to intracellular proteins or its receptors is the basis of a new strategy for the treatment of HCC and other cancers. In addition, AIFs can be combined with drugs and delivery agents to target treatments to cancer. AIFs conjugated to anticancer drugs not only destroy cancer cells with these drugs but also activate immune cells to kill cancer cells. Furthermore, AIF delivery of drugs relieves immunosuppression and enhances chemotherapy effects. The synergism of immunotherapy and targeted chemotherapy is expected to play an important role in enhancing the treatment effect of patients with cancer. AIF delivery of drugs will be an available strategy for the targeted treatment of cancer in the future.

## Introduction

Alpha fetoprotein (AFP) is an oncofetal protein that is highly expressed in fetal cells and in most patients with hepatocellular carcinoma (HCC), and it is a diagnostic marker of liver cancer (Bei and Mizejewski, 2011, 2020; Mizejewski, 2014, 2019; Bai et al., 2017; Kim et al., 2020; Mehta et al., 2020). Based on the origin, the types of AFP include natural AFP (nAFP), which is derived from fetal cells, and tumor-derived AFP (tAFP), which is highly expressed in HCC and other cancers. The forms of AFP are also categorized as secreted AFP (SeAFP) and cytoplasmic AFP (CyAFP) (Mizejewski, 2015a; Tcherkassova et al., 2017). Here, AFP mainly refers to tAFP, and CyAFP refers to cytoplasmic tAFP. The AFP amino acid sequence is highly homologous with albumin, and its structure is similar to that of albumin. However, the functions of AFP and albumin are different (Mizejewski, 2001, 2016). Albumin maintains stable plasma osmolality and delivers nutrients. AFP delivers nutrients, suppresses immunity and stimulates the growth of cancer cells. When the serum concentration of AFP is greater than 50 ng/ml in adult blood, it stimulates tissue regeneration or hematopoiesis, and it is also used by cancer cells to provide nutrients and stimulate growth (Mizejewski, 2002; He et al., 2014; Pak, 2018b).

Alpha fetoprotein regulates the expression of oncogenes, inhibits apoptosis, promotes cancer cell growth, enhances drug resistance, enhances the antitumor immune response, increases invasion, and increases metastasis, resulting in the malignant transformation of cancer, and these functions of AFP are referred to as AFP malignant behaviors (Meng et al., 2016; Lu et al., 2016; Suryatenggara et al., 2017; Komorowski et al., 2018; Mizejewski, 2019; Xue et al., 2020). AFP binds to its membrane receptor and cytoplasmic proteins to promote malignancy (Mizejewski, 2011b, 2014, 2015a, 2019; Pak, 2018b). Use of AFP-derived peptides, AFP fragments, and recombinant AFP (AFP-inhibiting fragments, AIFs) to prevent AFP from binding to signal transduction molecules or AFP receptors, thereby inhibiting the malignant behaviors mediated by AFP. Additionally, use of AIF conjugates with toxins or drugs to target its receptors to selectively destroy cancer cells (Mizejewski, 2011a, 2014; Pak, 2018b). Classically, AIF comprised of peptides and fragments which are derived from AFP domain-3. Here, we also categorize recombinant AFP (rhAFP) as an unique AIF because it can be used as a vector to deliver drugs to kill cancer cells. Peptide AIFs include AFP-derived growth inhibitory peptide (GIP) and its analogs. GIP does not bind the AFP receptor (AFPR), but it can enter the cells influence the enzyme activity of tumor cells (Mizejewski and Butterstein, 2006; Mizejewski et al., 2010). Other new peptides of AIF could be obtained from AFP domain-3, and these peptides can bind to AFPR or signal transduction molecules and serve as candidate decoy ligands to prevent malignancy mediated by AFP (Mizejewski, 2011b; Tcherkassova et al., 2017). Fragment AIFs include AFP-3BC, rAFP3D, and r3dAFP, which are fragments of protein derived from AFP domain-3, and they can deliver drugs and be endocytosed by cancer cells with high AFPR expression (Godovannyi et al., 2011; Posypanova et al., 2013; Yabbarov et al., 2013; Tcherkassova et al., 2017). The unique AIF, rhAFP is full-length AFP that is expressed in *E. coli* and in yeast cells (Arshad et al., 2015), and they can also be designed as candidate decoy ligands to deliver drugs to prevent AFP malignant behaviors. The reports on the use of AIFs for the treatment of cancer are shown in Table 1. In this review, we summarize the application of AIFs to deliver drugs for targeted cancer treatment.

**TABLE 1:** Reports of AFP-inhibiting fragments (AIFs) for the treatment of cancer.

**Name**	**Molecular derived**	**Binding drugs**	**The method of binding**	**References**
	**from AFP**			
GIP-34	It is a 34 amino acid peptide that derived from AFP domain-3			Mizejewski et al., 2010; Mizejewski, 2011b.
GIP-8	a peptide derived from AFP residues EMTPVNPG (AFPep)	Dox	Dox was synthesized by the use of a 4(4-N-maleimidomethyl)	Mizejewski et al., 2010; Mizejewski, 2011b.
			cyclohexane-1-carboxyl hydrazide crosslinker that forms a thioester bond between the 8-mer peptide and the Dox (GIP-8–Dox)	
GIP-P12	A synthetic peptide derived from the AFP domain-3			Mizejewski, 2011b.
AFP-3BC	recombinant fragment protein derived from AFP domain-3 (from 473–596 residues)	Dox	AFP-3BC was activated with SATA, and Dox was modified with EMCH, then Dox-EMCH and activated AFP-3BC were conjugated (Dox-AFP-3BC)	Posypanova et al., 2013
r3dAFP	recombinant fragment protein derived from C-terminal AFP (from 357–590 residues)	Dox, Paclitaxel	Dox or paclitaxel containing nanoparticles bound to r3dAFP (NP-Dox-r3dAFP NP-paclitaxel-r3dAFP)	Godovannyi et al., 2011; Posypanova et al., 2008
rAFP3D	recombinant fragment protein derived from AFP domain-3 (from 404–609 residues)	Dox	A three-component	Yabbarov et al., 2013
			delivery system including vector protein rAFP3D, polyamidoamine (PAMAM) generation 2 (G2) dendrimers and antitumor antibiotic Dox (rAFP3D-G2-Dox)	
Recombinant AFP (rhAFP)	full-length AFP gene were expressed in *E. coli* as well as in yeast cells	1′-S-1′- acetoxychavicol acetate (ACA), or other drugs	Non-covalent complexes of rhAFP and ACA or other drugs	Arshad et al., 2015

## Distribution and Function of the AFP Receptor in Cells

**FIGURE 1:**
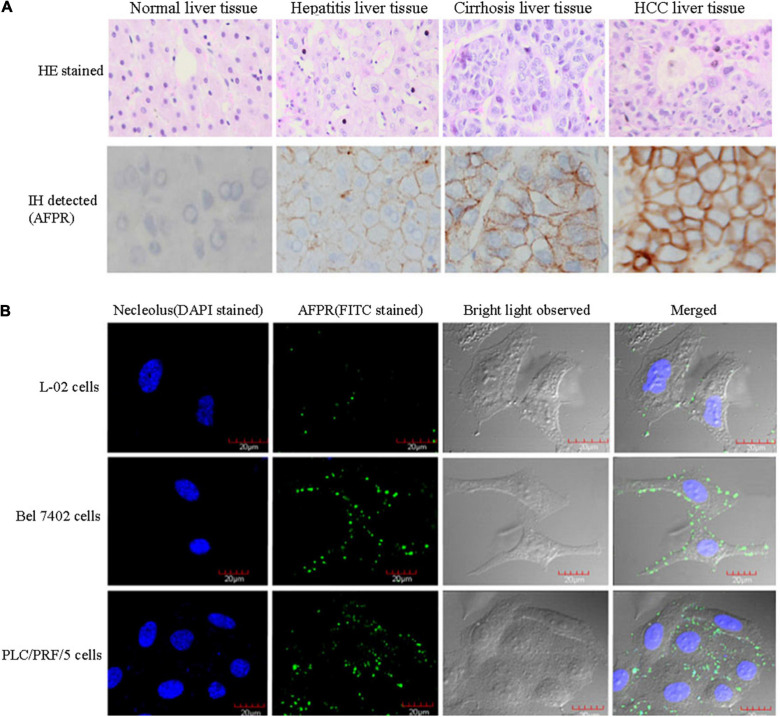
Expression of AFPR in clinical patient liver tissues and HCC cells. **(A)** Clinical liver tissue samples were collected after surgical hepatectomy, and the expression of AFPR in clinical patient liver tissues was detected by immunohistochemical assays. HE stained: hematoxylin-eosin stained; IH stained: immunohistochemical stained; red arrow indicates AFPR. **(B)** Expression and localization of AFPR (green fluorescence) in HCC cells were observed by laser confocal microscopy. Reprinted from Li et al. (2013) with permission from Elsevier.

Alpha fetoprotein is a shuttle protein that is endocytosed mainly upon binding to its receptor. Previously, many researchers have suggested that AFP might bind to cellular membrane proteins (Naval et al., 1985; Biddle and Sarcione, 1987; Torres et al., 1989; Mizejewski, 1995, 2013, 2014, 2019), and further analysis indicated that these proteins are receptors of AFP (Suzuki et al., 1992; Mizejewski, 2014, 2019). The AFP receptor (AFPR) is expressed in myoblasts (fetal cells) (Lorenzo et al., 1996), NIH3T3 cells, and malignant cells (Laborda et al., 1987; Esteban et al., 1991; Torres et al., 1991; Li et al., 2002c; Mizejewski, 2011b), but it is not expressed in well-differentiated myotubes (adult-like cells) (Lorenzo et al., 1996). Recently, we detected the expression and location of AFPR in normal liver cells and HCC cells by immunohistochemistry and laser confocal microscopy. High expression of AFPR has been observed in the membrane of HCC cells (Li et al., 2013; Zhu et al., 2015b; Figure 1). Two subtypes of AFPR have been identified in NIH3T3 and HCC cells with different Kds (Li et al., 2002a,c), suggesting that AFPR exists in at least two subtypes. AFP binds with AFPR, which increases the concentrations of cAMP and Ca^2+^ in the cytoplasm and promotes the expression of some oncogenes (Li et al., 2002c, 2004). Activation of growth and signaling pathways are pivotal factors by which AFP promotes hepatocarcinogenesis (Wang et al., 2012; Mizejewski, 2015a; Zhang et al., 2015, 2016; Xue et al., 2020). These results suggest that AFPR in the cellular membrane has traits of G protein-coupled receptors (GPCRs) and that the signal transduction of AFPR follows the principles of GPCRs. Secreted AFP has many functions, such as immunosuppression, and it regulates the malignant behaviors of cancer cells through mediation by AFPR (Mizejewski, 2018, 2019).

AFP was initially found to be taken up by fetal cells (Sell et al., 1985; Iturralde et al., 1991; Alava et al., 1999; Nierhoff et al., 2005), and later studies found that muscle tumor cells also internalize exogenous AFP (Uriel et al., 1983; Lorenzo et al., 1996; Mizejewski, 2011b, 2018). In addition, AFP binds to cellular membrane receptors in pits in the membrane bilayer, thereby triggering their internalization by cells. AFP and its receptor are packaged in endosomal vesicles and transported to the trans-Golgi network distributed near the nucleus (Lorenzo et al., 1996; Torres et al., 1991; Mizejewski, 2011b, 2018). Finally, the vesicles release AFP and its receptor complex into the cytoplasm where they are translocated to cytoplasmic organelles undergoing lysosomal degradation or engage in signal transduction pathways. For example, AFP binds to PTEN in the cytoplasm and activates PI3K signaling pathways, thus stimulating the growth of many malignant cells (Wang et al., 2012; Zhang et al., 2015, 2016; Mizejewski, 2019).

Although AFP-binding receptors are critical for receptor-mediated endocytosis and the uptake of AFP into the cytoplasm, the complete AFP-binding receptor structure has not yet been elucidated. Many AFP-binding receptors have been reported, and they are mainly classified into two categories as follows: (a) the mucin (MUC) family and (b) the scavenger receptor (SR) family (Uriel et al., 1984; Mizejewski, 2013, 2014, 2015b, 2019). Although the details of the AFP-binding receptor structure are not known, many studies have shown that cancer cells take up AFP through AFP-binding receptors (Laborda et al., 1987; Esteban et al., 1991; Torres et al., 1991; Mizejewski, 2011b, 2013, 2014, 2019; Zhu et al., 2015b).

Because HCC and other cancer cells regain the ability to take up AFP via its receptor and exert malignant behaviors (Li et al., 2013; Zhu et al., 2015b), AFP delivery of cytotoxins is used to target and kill cancer cells. It has been demonstrated that AFP is effective for drug delivery, but AFP, especially tumor-derived AFP (tAFP), is also immunosuppressive and thus can stimulate immune escape of cancer cells. AFP may also promote initiation of cancer. Therefore, it is better to use AIFs to deliver drugs to target cancer therapeutics (Mizejewski, 2011a; Pak, 2014, 2018b). Experiments with radioactively labeled an AIF (AFP-3BC) have confirmed that they selectively accumulate in cancer cells and that AFP-3BC loaded with drugs binds to human breast MCF7 cells and ovarian adenocarcinoma SKOV3 cells, suppressing the proliferation of these cancer cells. Importantly, AFP-3BC do not bind to non-stimulated lymphocytes. These findings indicate that AFP-3BC can be a promising new vector for selectively targeting and inhibiting the malignant behaviors of cancer cells (Posypanova et al., 2008; Mizejewski, 2011a; Posypanova et al., 2013).

## AFP Promotes the Malignant Behaviors of Cancer Cells

**FIGURE 2:**
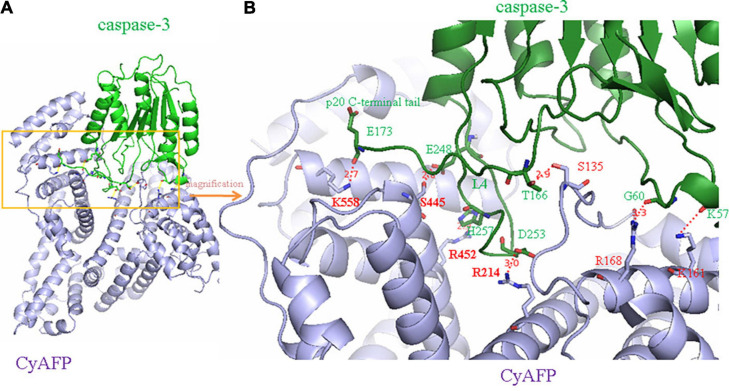
A molecular structure model of the interaction of CyAFP with caspase-3. **(A)** Overall structure of CyAFP in complex with caspase-3. CyAFP (blue) binds to caspase-3 (green) in the hydrophobic pocket. **(B)** Residues of CyAFP interact with caspase-3 (Lin et al., 2017).

Because AFP is a growth-promoting factor, it mostly promotes the growth of cancer cells. AFP binding to its receptors activates the cAMP-PKA pathway and induces Ca^2+^ influx, which promotes the expression of the *c-fos*, *c-jun* and *Ras* oncogenes and stimulates the growth of hepatoma cells (Li et al., 2002a,c; Ma et al., 2010; Wang et al., 2012; Zhang et al., 2012, 2015, 2016). In addition, after binding to receptors, AFP not only triggers growth-promoting signals but also stimulates the endocytosis of AFP into cells (Torres et al., 1991; Mizejewski, 2011b; Kong et al., 2012). The endocytosed AFP is released (which becomes CyAFP) from its receptor and then binds with some cytoplasmic proteins, leading to the activation or inhibition of signaling pathways. For example, CyAFP binding to caspase-3 inhibits the apoptosis signaling pathway (Li et al., 2009a; Lin et al., 2017). CyAFP binding to caspase-3 is shown in Figure 2. Caspase-3, also called cysteine aspartyl proteinase 3, plays an important role in the apoptosis pathway of cancer cells (Riedl et al., 2001; Zhang et al., 2019; Jiao et al., 2020). Activated caspase-3 protein binds to its substrate and induces apoptosis through cascade amplification, indicating that caspase-3 is the main executor of apoptosis (Mittl et al., 1997; Rogers et al., 2017). The binding of CyAFP to caspase-3 prevents apoptotic signal transduction in HCC cells. CyAFP not only directly binds to caspase-3 and inhibits its activity but also affects the activation of caspase-3 through the mitochondrial apoptosis pathway. Yang et al. (2008, 2018) found that blocking the expression of AFP increases the ratio of Bax/Bcl-2 and releases cytochrome C from the mitochondria, thus activating caspase-3 to induce apoptosis. These results suggest that CyAFP inhibits apoptosis in HCC through the Bax/cytochrome C/caspase-3 signaling pathway and promotes the proliferation of hepatoma cells. In addition, CyAFP also binds to the all-*trans* retinoic acid (ATRA) receptor, RAR-β, and inhibits receptor entry into the nucleus, leading to increased expression of apoptosis-inhibiting proteins, such as survivin (Li et al., 2009b; Zhang et al., 2020).

In addition, a study based on laser confocal microscopy, immunoprecipitation, fluorescence energy resonance transfer, molecular simulation and site-directed mutagenesis has shown that CyAFP also binds to PTEN, which is an important tumor suppressor that negatively regulates the PI3K/Akt signaling pathway (Li et al., 2011; Wang et al., 2018). PTEN binds to PI3K subunits, inhibits the phosphorylation of PI3K and blocks signal transduction by PI3K/AKT (Lee et al., 2018). Specifically, the CyAFP interaction with PTEN activates the PI3K/AKT/mTOR pathway, inhibiting autophagy and promoting the malignant behavior of HCC by upregulating the expression of mTOR protein. After CyAFP binds to PTEN, the regulatory function of PTEN is lost, which leads to the continuous phosphorylation of PI3K and activation of the downstream molecule, AKT, thereby leading to the malignant transformation of liver cancer cells (Li et al., 2011; Wang et al., 2018). Activated AKT stimulates the mTOR transcription cofactor and the STAT3 and HIF-1α transcription factors, which regulates the expression of oncogenes, inhibits apoptosis and inhibits autophagy in hepatoma cells as well as promotes the growth of cancer cells (Missiaglia et al., 2010; Li et al., 2011; Lee et al., 2018; Wang et al., 2018).

Cytoplasmic AFP binding to PTEN not only promotes the growth of liver cancer cells but also enhances the drug resistance of cancer cells. For example, CyAFP plays an important role in promoting the drug resistance of HCC (Li et al., 2012, 2020). The binding of CyAFP to PTEN activates the PI3K/AKT signaling pathway and interferes with the activity of caspase-3 (Zhu et al., 2015a,c), which leads to the drug resistance of hepatoma cells. A high concentration of CyAFP in liver cancer cells not only promotes growth but also results in a loss of sensitivity to drugs *in vivo* (Cheng et al., 2013; Li et al., 2020). The 2018 EASL clinical practice guidelines suggest that AFP can be used as an indicator for the diagnosis and prognosis of advanced HCC (European Association for the Study of the Liver, 2018). Many HCC patients with elevated SeAFP or CyAFP expression may have drug resistance and a poor prognosis.

## Design of AIFs From AFP Domain-3 and Applications for Targeting Deliver Drugs to Cancer Cells

**FIGURE 3:**
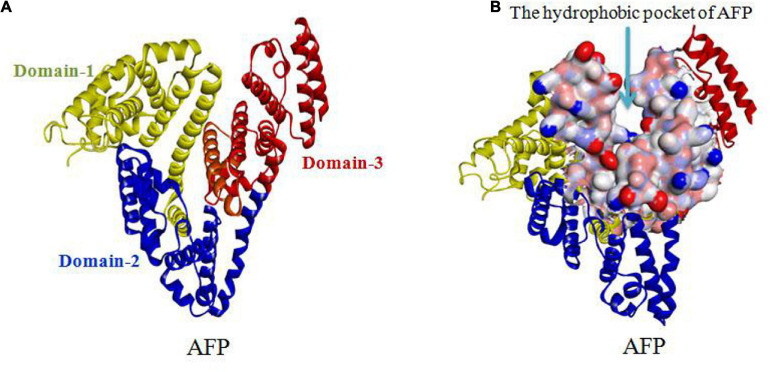
V-shaped AFP and its hydrophobic pocket. **(A)** Overall V-shaped structure of AFP comprised of three domains. Domain-1 (yellow) and domain-3 (red) are located on each side of the V-shape, and domain-2 (blue) is located at the bottom of the V-shape. **(B)** A hydrophobic pocket is formed between domain1 and domain-3 (Muehlemann et al., 2005; Pak, 2014; Mizejewski, 2015b, 2016).

Alpha fetoprotein has a molecular weight of 69 kDa and consists of a single peptide chain with 590∼609 amino acids and three domains. The N-terminal region of AFP, consisting of residues 1∼230, belongs to domain-1. The middle region of AFP, consisting of residues 230∼400, belongs to domain-2. The C-terminal region of AFP, consisting of residues 400∼609, belongs to domain-3. The overall AFP structure is V-shaped (see Figures 3A,B). Domain-1 (yellow) and domain-3 (red) are located on each side of the V-shape, and domain-2 (blue) is located at the bottom of the V-shape. A hydrophobic pocket is formed between domain-1 and domain-3, and it transports nutrients, such as fatty acids (Mizejewski, 2001, 2015b, 2016; Muehlemann et al., 2005).

**FIGURE 4:**
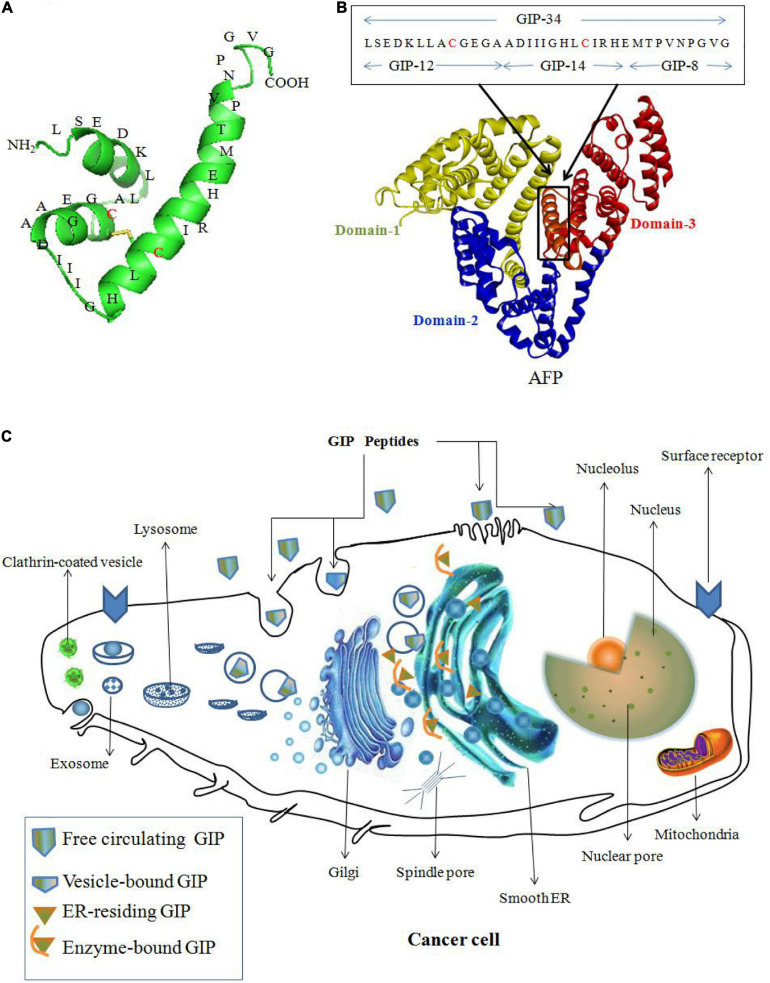
Peptide AIFs (GIP) derived from AFP domain-3 are taken up by cancer cells and prevent AFP-induced malignant functions. **(A)** Amino acid sequence of GIP-34. **(B)** Amino acid sequence of GIP analogs (GIP-8, 12, 14 and 34) derived from the AFP domain-3 sequence (Mizejewski et al., 2010). **(C)** The anticancer mechanism of GIP. GIP gains cell entry by: (1) direct cell membrane penetration; (2) channel formation; and/or (3) pore formation into the cell cytoplasm, and the cytoplasmic GIP target the smooth endoplasmic reticulum (ER) surrounding the nucleus and influence enzyme activity during the growth and metastasis of cancer cells (Mizejewski and Butterstein, 2006; Mizejewski et al., 2010; Mizejewski, 2011a).

Alpha fetoprotein domain-3 can be used to design peptide AIFs as vectors to deliver drugs to kill cancer cells. For example, GIP (Figures 4A,B) derived from domain-3 of the AFP sequence has the potential for treating cancer. GIP-34 and its analogs inhibit the migration and metastasis of cancer cells in both isograft and xenograft models. Additionally, GIP-34 and its analogs have been proposed to serve as vectors to deliver drugs to treat cancer, which will enhance the therapeutic effect (Mizejewski and Butterstein, 2006; Mizejewski et al., 2010; Mizejewski, 2011a). The anticancer mechanism of GIP may be explained as follows: GIP gains cell entry by: (1) direct cell membrane penetration; (2) channel formation; and/or (3) pore formation into the cell cytoplasm. And the cytoplasmic GIP influence enzyme activity which mediated by CyAFP during the growth and metastasis of cancer cells (Figure 4C). Such as GIP causes cancer cell growth suppression by inducing cell cycle arrest in the G1 to S-phase by preventing cell cycle p27 and p21 inhibitor degradations, thus halting cell cycle progression (Mizejewski and Butterstein, 2006; Mizejewski et al., 2010; Mizejewski, 2011a, 2013).

**FIGURE 5:**
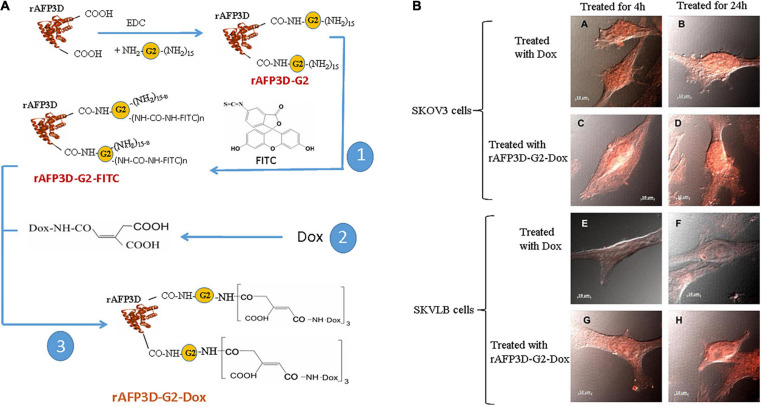
Scheme of conjugate synthesis of rAFP3D-G2-Dox and the accumulation of doxorubicin (Dox) in the drug-sensitive human ovarian adenocarcinoma SKOV3 cell line and the drug-resistant human ovarian adenocarcinoma SKVLB cell line. **(A)** rAFP3D conjugated to Dox (rAFP3D-G2-Dox). **(B)** Intracellular distribution of rAFP3D-G2-Dox and free Dox in (A∼D) the drug-sensitive human ovarian adenocarcinoma SKOV3 cell line and (E∼H) the drug-resistant human ovarian adenocarcinoma SKVLB cell line. Reprinted from Yabbarov et al. (2013) with permission from Elsevier.

As mentioned previously, the AFP domain-3 can also be used to design fragment AIFs conjugated with toxins. For example, Yabbarov et al. applied a fragment AIF (rAFP3D, which is designed from AFP domain-3) as a vector molecule conjugated to doxorubicin (Dox) (shown in Figure 5A) and utilized the drug-sensitive human ovarian adenocarcinoma SKOV3 cell line and the drug-resistant human ovarian adenocarcinoma SKVLB cell line to observe rAFP3D-Dox in these cells (Yabbarov et al., 2013). These researchers found that in drug-sensitive SKOV3 cells, there was little difference in the accumulation of Dox in the cytoplasm and nucleus when treated with free Dox or rAFP3D-Dox, but in the drug-resistant SKVLB cells, there was a significant increase in the accumulation of Dox in the cytoplasm and nucleus when treated with the rAFP3D-Dox compared to the control free Dox (Figure 5B). These results show that rAFP3D conjugated with fluorescein or Dox can be taken up by cancer cells, indicating that AFPR mediates AFP-derived rAFP3D-fluorescein or rAFP3D-Dox endocytosis into cancer cells and that rAFP3D-Dox induces cytotoxicity, resulting in cancer cell destruction. Thus, these studies indicate that rAFP3D can be applied in cancer treatment (Yabbarov et al., 2013).

Fragment AIFs can be used to prevent the AFP-mediated activation of proliferation-related signaling pathways to prevent drug resistance. Fragment AIFs can be conjugated with drugs to improve the sensitivity of cancer cells to agents (Godovannyi et al., 2011; Pak, 2018b). During cancer therapy, cancer cells may reduce their intake of anticancer drugs, such as methotrexate, paclitaxel, anthracyclines, platinum derivatives, 5-fluorouracil (5-FU), gemcitabine, capecitabine and sorafenib (Godovannyi et al., 2011; Pak, 2014), which may lead to decreased or inactivated drug sensitivity. However, these drugs can be conjugated to fragmented AIFs. Because fragment AIFs, such as rAFP3D, recognize and bind to AFP receptors on the membranes of cancer cells, they transport drugs into the cell through receptor-mediated endocytosis, which increases the intake of drugs and enhances the accumulation of drugs, allowing the drugs to exert their cytotoxic effects. For example, rAFP3D conjugated to Dox (rAFP3D-G2-Dox) increases the sensitivity of human ovarian carcinoma cells, breast cancer cells and other cancer cells to Dox (Godovannyi et al., 2011; Yabbarov et al., 2013; Tcherkassova et al., 2017).

**FIGURE 6:**
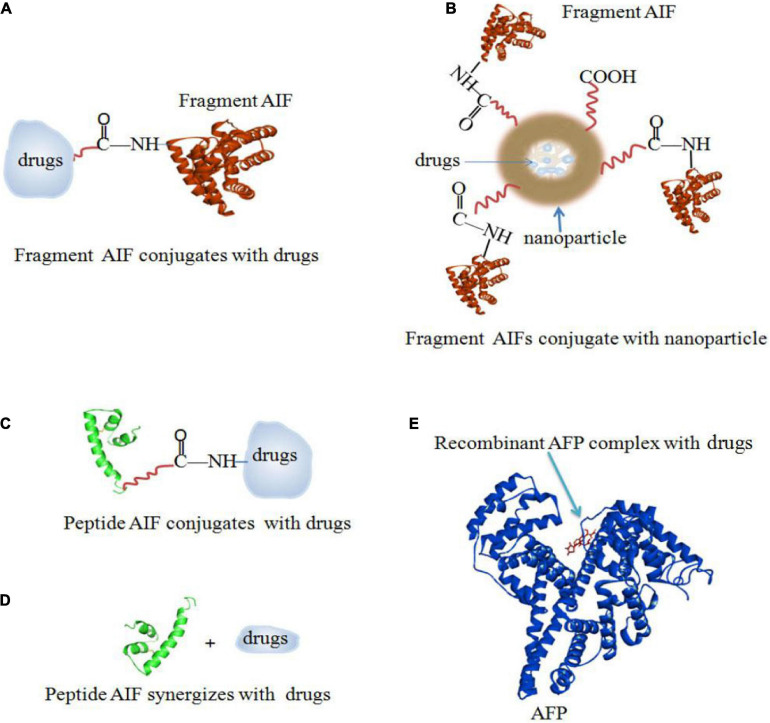
AIFs (fragment AIFs, peptide AIFs and recombinant AFP) conjugate, synergize and complex with drugs. **(A)** Fragment AIF conjugates with drugs (Posypanova et al., 2013; Yabbarov et al., 2013). **(B)** Fragment AIFs conjugate nanoparticles with drugs (Godovannyi et al., 2011; Yabbarov et al., 2013). **(C)** Peptide AIF conjugates with drugs (Mizejewski et al., 2010). **(D)** Peptide AIF synergizes with drugs (Mizejewski et al., 2010). **(E)** Recombinant AFP complex with drugs (Arshad et al., 2015; Pak, 2018b).

There are several ways to conjugate AIFs with drugs. Figure 5A shows a method of rAFP3D conjugation to Dox (rAFP3D-G2-Dox) (Yabbarov et al., 2013; Posypanova et al., 2013). rAFP3D can also link nanoparticles and liposomes to increase the effectiveness of targeted therapy (Figures 6A,B; Godovannyi et al., 2011; Yabbarov et al., 2013). Other AIFs, such as peptide AIFs, can conjugate or synergize with drugs to treat cancer (Figures 6C,D). Recombinant AFP (a unique AIF) may be designed to retain the hydrophobic pockets of AFP, which may non-covalently bind to a variety of drugs and effectively release them inside cancer cells (Figure 6E) (Mizejewski et al., 2010; Arshad et al., 2015; Pak, 2018b).

## Application of AIF to Enhance Immunotherapy of Cancer

Cancer cells can be produced *in vivo* at any time, but the immune system eliminates them quickly and maintains a healthy state. Mutations make cancer cells different, and immune cells can recognize cancer cells based on certain differences and attack them (Marx, 2008; Mohme et al., 2017). However, cancer cells can acquire skills to promote their own survival (DuPage et al., 2012; Ribas, 2015; McGray and Bramson, 2017). For example, cancer cells can mask proteins to prevent immune cells from recognizing them or produce proteins that suppress immunity to escape immunity without being attacked. Thus, cancer cells can survive and proliferate *in vivo*. As cancer cells proliferate and continue to evolve *in vivo*, it becomes increasingly difficult for the immune system to attack them.

Natural killer (NK) cells, which have a natural immune function, are the first-line defense system. NK cells can prevent cancer cell growth, invasion and metastasis as well as attack pathogens. NK cells constantly surveil and eliminate cells that pose a threat to health. Disruption of NK cell action can lead to diseases or carcinogenesis (Shimasaki et al., 2020). Similarly, immune T cells *in vivo* can monitor and eliminate cancer cells. However, cancer cells can disguise themselves through the production of myeloid-derived suppressor cells (MDSCs) and evade the surveillance of the immune system (Dumitru et al., 2012; Baniyash, 2016). MDSCs express proteins that bind with proteins on immune cells and signal them to “turn off” their immune functions (Bruno et al., 2019). MDSCs are advanced immunosuppressive cells that are produced from the bone marrow and transported to the primary lesion where they accumulate and suppress acquired immunity and innate immunity. In cancer, the immune system produces MDSCs from the bone marrow, and they proliferate in the blood and normal peripheral organs. Further, cancer cells develop immune tolerance.

Targeting MDSCs is a new approach to immunotherapy that can eliminate immune tolerance molecules, activate NK cells, activate T cells and engage the immune system in recognizing and destroying cancer cells through a positive response (Makkouk and Weiner, 2015; Pak, 2017). Many lines of evidence have indicated that AFP inhibits the immune response in patients with cancers (Um et al., 2004; Meng et al., 2016; Suryatenggara et al., 2017; Wang and Wang, 2018; Zheng et al., 2020). AFP inhibits the activity of NK cells and T cells by activating AFPR-positive MDSCs and promoting cancer development (Belyaev et al., 2018; Zamorina et al., 2018). Therefore, vaccines against AFP inhibit the growth of AFP receptor-positive cancer cells and prolong patient survival time (Lan et al., 2007). One study has reported that inoculation of the placental carcinoembryonic-derived proteins, AFP and AFPR, causes MDSCs to become exhausted, resulting in the elimination of maternal-fetal and host-tumor immune tolerance (Mizejewski, 2018; Pak, 2018b). Thus, an AFP vaccine promotes a longer survival of advanced patients with cancer. In some cancers, full-length glycosylated AFP has immunosuppressive effects by stimulating cancer growth and directly activating MDSCs (Pak, 2018a,b). Moreover, tAFP significantly inhibits dendritic cell (DC) differentiation, thereby playing a critical role in immunosuppression (Pardee et al., 2014; Li et al., 2019). Therefore, it is more suitable to use AIF than tAFP for manufacturing vaccines to prevent the initiation of cancer.

Currently, the main research direction of immunotherapy involves immune checkpoint inhibitors as represented by treatment with PD-1/PD-L1 inhibitors. PD-1/PD-L1 inhibitors prevent PD-L1 from binding to PD-1 on immune cells, relieving the inhibition of cancer immunosuppression and stimulating immune cells to attack cancer cells (Chen and Han, 2015; Postow et al., 2015; Sun et al., 2018; Andrews et al., 2019; Hayashi and Nakagawa, 2020). In patients with HCC, high expression of both PD-L1 and HIF-1α is significantly associated with high AFP levels (Dai et al., 2018; Liu G. M. et al., 2019), indicating that the expression of AFP is closely related to the expression of PD-L1 in HCC cells. The expression of PD-L1 is regulated by the HIF-1α transcription factor (Koh et al., 2015; Chen et al., 2016; Zerdes et al., 2018). Researchers have previously reported that AFP activates the PI3K/AKT signaling pathway to stimulate HIF-1α, which regulates the expression of some oncogenes (Zhu et al., 2015a,b), indicating that AFP has a biological role in stimulating the expression of PD-L1 in cancer cells. In HCC cells, the persistent expression of PD-L1 in HCC cells is a crucial factor for resisting immune checkpoint inhibitors (Liu Z. et al., 2019; Wu et al., 2019). We speculate that high AFP expression in HCC cells promotes HIF-1α to stimulate the expression of PD-L1, which plays a pivotal role in HCC cells resisting immune checkpoint inhibitors. Therefore, AIFs can be used to carry PD-1/PD-L1 inhibitors because AIFs do not induce the malignant behavior caused by tAFP. AIFs exhaust MDSCs and cancer cells, and PD-1/PD-L1 inhibitors reactivate the function of T cells, which leads to the activation of NK cells, restoring their normal function of recognizing cancer cells and destroying them.

In addition, synergistic immunotherapy with chemotherapy is better for treating cancer. AFP activates MDSCs and inhibits various non-specific immune reactions (Pardee et al., 2014; Belyaev et al., 2018). The structure of AFP-binding receptors is still unclear, but MDSCs and cancer cells have ‘scavenger’ receptors that are similar to AFP-binding receptors and are critical for receptor-mediated endocytosis. The receptors take up AFP and provide nutrients to cancer cells and bone marrow mesenchymal stem cells through shuttling; thus, AFP delivery of drugs instead of nutrients kills MDSCs and cancer cells. The use of AIFs combined with toxicity-inducing drugs is a new treatment that integrates chemotherapy and immunotherapy. AIFs can deplete MDSCs, and inhibiting fragments loaded with toxins can destroy cancer cells. For example, AIFs conjugated with paclitaxel, 5-Fu or other chemotherapeutic drugs not only are used as toxins to kill cancer cells but also serve as immunomodulators. AIFs conjugated with drugs selectively reduce the immunosuppression of MDSCs and destroy cancer cells to improve the treatment of cancers.

The conjugation of AIFs with drugs is a new type of treatment for cancer. It not only activates T cells and kills cancer cells by drugs but also depletes MDSCs, activates NK cells and destroys cancer stem and metastatic cells through AIFs. This combination also activates T cells through drug action. Conjugating AIFs and drugs is a new approach to immunotherapy and targeted chemotherapy, and this combination will play an important role in future cancer therapies (Mizejewski, 2011a; Pak, 2014, 2018b; Llovet et al., 2018; Pinter and Peck-Radosavljevic, 2018).

## Forecasts of the Design and Application of AIFs in Targeting Therapeutics of Cancers

Cancer cells with multi-drug resistance (MDR) traits resist chemotherapy, and they express PD-L1 to suppress the immune response and escape immune surveillance, preventing the attack of immune cells (Ribas, 2015; Berraondo et al., 2016; Chen and Mellman, 2017; O’Donnell et al., 2019). tAFP is a crucial molecule for promoting the malignant behaviors of HCC cells, primarily by activating growth signaling pathways. Other cancer cells also take up AFP to activate malignant signaling pathways to acquire drug resistance, contributing to their survival *in vivo*. tAFP also has the capacity to impair immune cells (Bei et al., 1999; Pardee et al., 2014; Vujanovic et al., 2017; Santos et al., 2019) and stimulate the malignant behaviors of cancer cells. Therefore, it will better to design AIFs to prevent the malignant behaviors mediated by tAFP in cancer cells.

In previous studies, we found that the cytoplasmic tAFP (CyAFP) binds to caspase-3, ATRA, PTEN and other proteins (Li et al., 2002b, 2009a, 2011, 2012; Zhu et al., 2015c; Lin et al., 2017; Wang et al., 2018; Zhang et al., 2020) to affect the transduction of apoptosis- or proliferation-related signaling pathways. CyAFP binding to caspase-3 is shown in Figure 2. In particular, we found that the CyAFP domain-3 residues, K-558, S-445, R-452 and its adjacent residue R-214, directly interact with caspase-3 loop4 (L4) residues in the cytoplasm (Lin et al., 2017). Through these binding sites, a peptide or fragment AIF can be precisely designed as a ligand decoy, which will prevent the binding of CyAFP and caspase-3, thereby promoting cell apoptosis. Similarly, we found that the CyAFP domain-3 residues, M490 and D529, interact with PTEN (Zhu et al., 2015c). Based on the binding sites, relevant AFP-blocking peptides can be designed to inhibit AFP binding to PTEN, which will prevent the growth of cancer cells. In addition, cancer therapy realized by targeting AFP may overcome the problem of MDR. MDR is a major problem that vexes clinical oncologists. Although the MDR mechanism in cancer is complicated, studies have found that AFP is involved in MDR by inhibiting the function of PTEN and activating the PI3K/AKT signaling pathway, which leads to the inhibition of autophagy, induction of metabolic reprogramming of cancer stem cells, inhibition of the expression of apoptosis-related enzymes and resistance to tumor cell apoptosis, thereby enabling cancer cells to acquire a drug-resistant phenotype (Kang-Park et al., 2006; Fruman et al., 2017; Zhu et al., 2017; Janku et al., 2018; Hoxhaj and Manning, 2020).

Cytoplasmic tAFP domain-3 (CyAFP-3D) is a pivotal site for inhibiting PTEN and caspase-3 (Li et al., 2009a; Mizejewski, 2015b; Zhu et al., 2015c; Lin et al., 2017; Wang et al., 2018; Li et al., 2020), leading to MDR. Therefore, CyAFP-3D can be used to design AIF for interacting with signaling molecules that play crucial roles in inhibiting immune responses and cancer cell growth, drug resistance and metastasis. The designed AIF can bind to intracellular caspase-3, PTEN and other signaling molecules to prevent AFP from binding to them and activating malignant signaling pathways.

Cytoplasmic AFP domain-3 can also be used to design AIFs to deliver drugs to target cancer cells. Because some cancer cells and immune suppressive cells have high expression of AFPR *vs* normal cells, AIF will bind to AFPR and transport drugs into cancer cells and immune suppressive cells, thereby resulting in low cytotoxicity in normal cells. Therefore, a precisely designed AIF can be used to block AFP-stimulated malignant behavior and to carry anticancer drugs to selectively treat cancers (Godovannyi et al., 2011; Posypanova et al., 2013; Yabbarov et al., 2013; Tcherkassova et al., 2017).

Alpha fetoprotein-inhibiting fragments also competes with CyAFP in immune cells to decrease the immune suppression mediated by CyAFP. Additionally, AIF can be designed to block CyAFP from activating the transcriptional activity of HIF-1α, which regulates the expression of PD-L1 in cancer cells, thus contributing to immune cells attacking cancer cells.

## Conclusion and Future Perspectives

Alpha fetoprotein-inhibiting fragments selectively deliver antineoplastic agents to cancer cells to inhibit the malignant behaviors mediated by CyAFP, representing a precise design for targeting and killing cancer cells. Moreover, blocking the immunosuppressive effect of CyAFP is a crucial issue for stimulating the immune response to cancer cells. CyAFP promotes the malignant behaviors of cancer cells and impairs the function of immune cells. Domain-3 of CyAFP can be applied to precisely design AIFs to carry anticancer drugs to selectively accumulate them in cancer or immunosuppressive cells. Precisely designed AIFs not only deliver drugs into cancer cells but also compete with CyAFP to bind to various signaling molecules, inhibiting the role of CyAFP in promoting the malignant behaviors of cancer cells and blocking its effect on immunosuppression. These AIFs can be combined with immunotherapy drugs to strengthen the therapeutic effect. In the future, computer simulation screening will be used to establish a database of AIFs that are effective in treating cancer and a database of drugs that can be conjugated with AIFs. Therefore, the application of AIFs will be a precise, readily available strategy for targeted treatment of cancers in the future.

## Author Contributions

BL, XD, and QW gathered the related literature, prepared the figures, and drafted the manuscript. WL, MZ, and ML participated in the design of the review and drafted the manuscript. All authors read and approved the final manuscript.

## Conflict of Interest

The authors declare that the research was conducted in the absence of any commercial or financial relationships that could be construed as a potential conflict of interest.

## Abbreviations

AFP: alpha fetoprotein
AFP-3BC: recombinant fragment protein derived from AFP domain-3 (from 473 ∼ 596 residues)
AFPR: AFP receptor
AIFs: AFP-inhibiting fragments
ATRA: all-transretinoic acid
CyAFP: cytoplasmic AFP
DC: dendritic cell
Dox: doxorubicin
GIP: AFP-derived growth inhibitory peptide (synthetic growth inhibitory peptide derived from AFP domain-3)
GPCRs: G protein-coupled receptors
HCC: hepatocellular carcinoma
HIF-1 α: hypoxia-induced factor (HIF)-1 α
Kds: dissociation constants
MDR: multi-drug resistance
MDSCs: myeloid-derived suppressor cells
nAFP: natural AFP (derived from fetal cells)
NK: natural killer
PTEN: phosphatase and tensin homolog gene deleted on chromosome 10
r3dAFP: recombinant fragment protein derived from C-terminal AFP (from 357 ∼ 590 residues)
rAFP3D: recombinant fragment protein derived from AFP domain-3 (from 404 ∼ 609 residues)
RAR: retinoic acid receptor
rhAFP: recombinant AFP (full-length AFP gene was expressed in *E. coli* as well as in yeast cells which had biological properties related to but not identical to native human AFP)
SeAFP: secreted AFP
tAFP: tumor-derived AFP
